# Parallel Changes in Cognitive Function and Gray Matter Volume After Multi-Component Training of Cognitive Control (MTCC) in Adolescents

**DOI:** 10.3389/fnhum.2019.00246

**Published:** 2019-07-16

**Authors:** Dasom Lee, Seyul Kwak, Jeanyung Chey

**Affiliations:** Department of Psychology, Seoul National University, Seoul, South Korea

**Keywords:** cognitive control, cognitive training, inhibition, working memory, shifting, voxel-based morphometry, inferior frontal, brain plasticity

## Abstract

Adolescence is a unique period in which higher cognition develops to adult-level, while plasticity of neuron and behavior is at one of its peak. Notably, cognitive training studies for adolescents has been sparse and neural correlates of the training effects yet to be established. This study investigated the effects of multi-component training of cognitive control (MTCC) in order to examine whether the training enhanced adolescents’ cognitive control ability and if the effects were generalizable to other cognitive domains. Cognitive control refers to the ability to adjust a series of thoughts and behaviors in correspondence to an internal goal, and involves inhibition, working memory, shifting, and dual tasking as subcomponents. The participants were middle school students (aged 11–14) and randomly assigned to either a training group or an active control group. The training group performed 30 min of MTCC per day for 6 weeks. To identify the training effects, we examined the cognitive performance, regional gray matter, and their relationship. The training group showed modest improvement in a visuospatial fluid intelligence test (Block Design) after MTCC, which was not significant after correcting for multiple comparisons. In addition, the training effect on the gray matter volume (time × group interaction) was observed in the right inferior cortex (rIFC). While the control group showed a typical reduction in the rIFC volume, the training group showed a relative increase in the homologous region. The relative change in rIFC volume was associated with the change in Stroop performance. These results imply that MTCC may affect brain structure relevant to inhibitory control process.

## Introduction

Cognitive control is an intentional top-down cognitive process that suppresses prepotent, automatic, and habitual behaviors to achieve a goal-directed behavior (Miller and Cohen, 2001; Diamond, 2013; Friedman and Miyake, 2017). This is an essential ability in self-regulation (Hofmann et al., 2012) and other cognitive functions such as memory, reading, and processing speed (Lustig et al., 2006; Blair and Razza, 2007; Healey et al., 2010). In addition, the development of cognitive control capacity continues until late adolescence or early adulthood (Casey et al., 2000; Diamond, 2002; Luna, 2009; Crone and Steinbeis, 2017). In terms of trainability, several studies of cognitive control training for clinical groups or the elderly (for example, Dahlin et al., 2008; Kray et al., 2012; Kim et al., 2017) have shown that this ability could be improved through training.

Recently, the need for cognitive control training in adolescence has been increasing. Cognitive control ability improves rapidly during this period approaching adult performance (Davidson et al., 2006) but with vast individual differences in developmental pace and trajectory. Further, cognitive and neural plasticity are relatively high in adolescence, compared with older age groups (Casey et al., 2005; De Magalhães and Sandberg, 2005). Therefore, the effects of cognitive training in adolescence can be expected to be substantial. In addition, this ability is required in successful academic achievement (Gathercole et al., 2004; Borella et al., 2010). As many more students pursue higher education globally, the ability to suppress habitual or irrelevant behaviors while promoting goal-directed behaviors is becoming important. Until now, however, cognitive training studies for adolescents have been limited to adolescents with neurodevelopmental disorders, such as attention-deficit/hyperactivity disorder (ADHD) (King et al., 2007) and autism spectrum disorders (Solomon et al., 2008), while those for typically developing adolescents have been scarce (Karbach and Unger, 2014).

According to the process-based training paradigm (Lustig et al., 2009; Morrison and Chein, 2011; Jolles and Crone, 2012), practicing a demanding cognitive task improves the efficiency of the cognitive processes, and increased efficiency can contribute to improved cognitive performance in an untrained task. Based on the experimental paradigms such as the Simon (Simon and Wolf, 1963) and the Flanker (Eriksen and Eriksen, 1974) tasks, we designed the cognitive training program to engage cognitive control processes. Cognitive control is hypothesized to comprise a set of processes such as response inhibition, updating, task switching, and coordinating multiple operations simultaneously (Buitenweg et al., 2012). Yet, high correlations between tasks provide evidence for the existence of a common latent factor (Emerson et al., 1999; Miyake et al., 2000; Friedman and Miyake, 2017). For example, the results of factor analysis (Friedman and Miyake, 2017) imply that inhibition may be included as part of a more general factor, rather than as a distinct factor. When performing working memory tasks, for instance, the task requires inhibition of the previous information irrelevant to the present goal.

In this study, we incorporated multiple subcomponents into the training, which could repeatedly access and enhance the more general component of the cognitive control function. Rather than repeating a single task numerous times as a training paradigm, the literature has demonstrated the effectiveness of a multi-component training approach that involves various cognitive components and modalities (Cheng et al., 2012; Gates and Sachdev, 2014; Kim et al., 2017).

Most studies examining the training effect have utilized change in cognitive performance as the index of the training. Despite its importance, investigating only cognitive performance limits the study in understanding the relevant neural change following cognitive training. Cognitive process is often specialized in a certain brain area which also has local plasticity (Zatorre et al., 2012). Evidence has indicated that intensive repetition of a specific cognitive process induces the functional and structural changes in brain regions specialized for the given tasks. For example, neural activities and gray matter (GM) volume in the right inferior frontal gyri significantly changed after a 2-week training of inhibitory control task (Chavan et al., 2015). In addition, a relatively large number of working memory training studies have also demonstrated significant changes in brain, such as functional activation or cortical thickness after training (Takeuchi et al., 2011; Román et al., 2015; Constantinidis and Klingberg, 2016). The literature suggests the necessity of examining the structural changes in the brain to explain the underlying mechanism of cognitive training effects.

Furthermore, when examining the training effect in the context of a developing brain, an additional challenge must be dealt with. Because adolescents are undergoing rapid brain maturation, disambiguating the training effect from the typical brain development can be challenging. For example, most of the previous studies conducted without control group cannot ensure whether the observed difference between the pre- and post-training sessions was related to the training or development (Jolles et al., 2010, 2012; Chavan et al., 2015). Further, even studies with control group involving either a passive or other different activity (e.g., waiting for a treatment, watching an educational video, and puzzle) might allow participants to identify themselves as a member of the control group. Therefore, the current study included an active control group to better identify the training effect by group comparison and also control for the placebo effect.

In this study, we investigated whether the multi-component training of cognitive control (MTCC), previously developed for the elderly adults (Kim et al., 2017), can enhance adolescents’ cognitive control ability and if these effects can be generalized to other cognitive domains. In order to make the training suitable for adolescents, we adjusted the difficulty level as well as the visual aspects of the stimuli so that it could be more challenging and meaningful for the age group. We administered a battery of neuropsychological tests before and after the training and explored the structural changes in the brain after the training to identify the neural basis of MTCC. We hypothesized that the changes would mainly occur in brain regions critically involved in cognitive control process that our MTCC program tapped into. We also hypothesized that brain structural changes track the individual difference in cognitive enhancements.

## Materials and Methods

### Participants

Sixty-four adolescents were recruited through a middle school, a scholastic academy and community websites. All the participants were right-handed, and exclusion criteria were based on a history of traumatic brain injury, neurodevelopmental disorders such as ADHD, or contraindication to MRI. We introduced non-adaptive, non-demanding training for the control group to exclude the placebo effect from the results. All the participants were randomly assigned to either a training group or control group by matching age and sex; among these, three dropped out of the study and 8 (1 control group, 7 training group) were not able to complete the 80% of the training course. Only those participants who completed more than 80% of the training session were included in the analysis to ensure that they achieved the minimum amount of training.

The remaining 53 participants (training group = 25, control group = 28) received and completed the adaptive or non-adaptive cognitive control training for 6 weeks, and their data were included in the analysis. No significant differences were observed regarding age, sex, vocabulary (scaled score) and test–retest interval between the two groups (Table 1). Cognitive assessment and MRI acquisition were administered before and after the training to investigate the effects of training. The study was approved by the Institutional Review Board of Seoul National University, and all the participants and their legal guardians were provided written consent for participation.

**Table 1:** Demographic statistics.

	Training Group (*n* = 25)	Control Group (*n* = 28)	t/χ^2^	*p*
	Mean (SD)	Mean (SD)		
Age	13.22 (0.71)	13.15 (0.58)	-0.36	0.721
Sex (Male:Female)	14:11	16:12	<0.01	1.000
Vocabulary	14.20 (2.36)	13.36 (2.88)	1.17	0.25
Test–retest interval (days)	68.56 (8.98)	71.71 (12.64)	-1.04	0.305

### Cognitive Training

Multi-component training of cognitive control (MTCC) was originally developed in-house at the Clinical Neuroscience Lab of Seoul National University (Kim et al., 2017), which was modified for adolescents in this study. MTCC was provided for 6 weeks through a computer in each participant’s home. The MTCC for adolescents comprised seven tasks: Location–Number Stroop, Rock–Paper–Scissors, Star and Moon, three versions of Updating (color, location, and letter), and Counting Clovers. Each task contained more than one component of cognitive control including the common factor heavily involving inhibition. More specifically, Rock–Paper–Scissors and Updating for working memory, Rock–Paper–Scissors and Star and Moon for shifting, and Counting Clovers for dual tasking. The training group was trained for 30 min a day, 5 days a week for 6 weeks. On each session, participants performed three tasks, each for 10 min, of which the list was pre-defined. Each task started at level one, and the level of each task continually increased to maintain a consistent cognitive load. If the accuracy increased to greater than 85%, the participants moved to the next level; if the accuracy level decreased to less than 65%, the participants returned to the previous level. The adherence to the training was confirmed daily through the recorded response time and accuracy for each task. If the response time were excessively long, or recorded answers were repeatedly incorrect, the training manager contacted the trainee to explain the task again and confirmed that they understood the task correctly.

#### Location–Number Stroop

In levels 1 and 2, a 4 × 4 or 5 × 5 matrix written with one number at each row appeared on the screen. The participants must respond to the location of a number, not the number itself. In levels 3 and 4, two 4 × 4 or 5 × 5 matrices appeared on the screen, and the participants must transpose the location of a number to another matrix. Location–Number Stroop required inhibition because the participants must suppress the number itself and respond to the location.

#### Rock–Paper–Scissors

The participants played Rock–Paper–Scissors according to the color of the hand. Four colors were used in this task: pink, blue, yellow, and green. The participants must beat the computer in the pink hand condition, lose in the blue hand condition, apply the same rule with the previous trial in the yellow hand condition, and apply the opposite rule with the previous trial in the green hand condition. As the level progressed, the color of the hand and the given rule became more complex (from pink to green). Rock–Paper–Scissors tapped the inhibition (suppressing original rule), updating (memorizing each rule), and shifting (response according to the color of hand) abilities.

#### Star and Moon

Four stimuli, a combination of two colors (yellow and blue) and two shapes (star and moon), were presented one by one. Star and Moon comprised two parts to train the set shifting. In part 1, the participants must respond to the yellow moon and blue star. In part 2, the participants must respond if a shape was different from the previous stimuli irrespective of colors. This task required shifting to flexibly change the rule.

#### Updating (Color, Location, and Letter)

Serial colors, locations, or letters appeared on the screen one at a time and the participants must memorize the last *n* colors, locations, or letters according to the levels. The participants did not know the number of stimuli presented; thus, the ability to update new information or working memory was involved.

#### Counting Clovers

A group of green and orange clovers appeared on either the right or the left side of the screen. The participants were required to press the right button if there were more green clovers and press the left button if more orange clovers were present. The color (green or orange) and side (right or left) of the clovers were chosen at random. Thus, the participants must overcome Simon interference if the location of color and button were inconsistent. The additional rule was introduced to train dual tasking. If the total number of clovers were 3, 5, 7, 9, or 11, the participants must press the spacebar on the keyboard.

To control the placebo effect, only the easiest level were provided to the control group within four training tasks. In the case of Location–Number Stroop and Rock–Paper–Scissors, the tasks were the same as level 1 of the training group tasks. Star and Moon were accomplished without shifting elements, that is, participants performed only part 1. Counting Clovers were also done without additional rule and the Simon interference. The right screen always showed green clovers, and the left screen always showed orange clovers, so participants only needed to respond according to the location where more clovers were presented. These simple control tasks shared the stimuli and rules but required minimum working memory, inhibition and interference components. Also, two tasks (5 min each) were provided in a single session, and the total assignment of the training time was minimally controlled (10 min × two sessions a week), which was only 1/20 amount of the training group. Thus, we expected to observe no or minimal training effects in the active control group.

### Cognitive Assessment

A battery of neuropsychological tests were administered to assess the effects of training. Assessment consisted of the Stroop color–word interference (Shin and Park, 2007) and the Trail Making Test (TMT) in the Korean version of the Consortium to Establish a Registry for Alzheimer’s disease (CERAD-K; Lee et al., 2002) for cognitive control; Symbol Span (SSP) in the Korean-Wechsler Memory Scale-IV (K-WMS-IV; Chey et al., 2011), Digit Span (DS) in the Korean-Wechsler Adult Intelligence Scale-IV (K-WAIS-IV; Hwang et al., 2012), and Arithmetic in Korean-Wechsler Intelligence Scale for Children-IV (K-WISC-IV; Kwak et al., 2011) for working memory; Verbal Paired Associate (VPA) and Design (DE) in K-WMS-IV for episodic memory; and Block Design (BD) and Matrix Reasoning (MR) in K-WISC-IV for fluid intelligence.

Three participants could not complete the TMT because they did not know the sequence of the Korean alphabet. In addition, one missing data point was observed for Stroop color and color–word test because of a color perception problem. All missing data were imputed using the aregImpute function in the Hmisc package (Harrell, 2018) in R. Each missing data point was predicted by all other non-missing cognitive test scores using bootstrap and predictive mean matching.

### Image Acquisition

Structural MRI data were collected to explore the training-induced changes in brain volume and cortical thickness. T1-weighted MPRAGE images were acquired by using a 3-Tesla MAGNETOM Trio 32 channel coil (sagittal slices, slice thickness 1 mm, TR = 2,300 ms, TE = 2.36 ms, FOV = 256 × 256 mm, FA = 9°, voxel size 1 mm × 1 mm × 1 mm). All images were visually inspected and rescanned when head motion confounded the image quality. The mean time interval between the pre- and the post-training was 65.23 days (*SD* = 9.65).

### Statistical Analyses

#### Behavioral Analysis

Before comparing the training group to the control group, group differences at baseline were examined by using an independent *t*-test and chi-square cross tabulation analysis. Next, individual training-performance change over time was investigated to examine the improvement in the training tasks and to ensure the quality of the training by using the lme4 (Bates et al., 2015) and lmerTest (Kuznetsova et al., 2017) package in R. The training performance was measured by the level and accuracy of the administered tasks and these data were fitted to a linear mixed-effects model (LMM) using maximum likelihood. The accuracy and maximum levels obtained by each participant during each training session were expected to show improvements if the training procedure was properly applied. Twenty-five participants completed the training program for 6 weeks. Among these, 22 participants’ training gains were analyzed because three participants’ data were missing due to technical problems. In the case of the control group, the improvement in the training task was not examined since the assigned tasks showed a ceiling effect in the first session.

In the next step, difference scores between pre- and post-assessments were tested by using a paired *t*-test and repeated-measure analysis of variance (ANOVA) to evaluate the effects of the MTCC. In the case of Stroop and TMT, the interference index was calculated by comparing the conditions. More specifically, the Stroop interference score (IG) was calculated by using the following formula proposed by Golden (1978); “IG = CW – Pcw” (where Pcw = (W × C)/(W + C), W: word, C: color, CW: color–word condition). The TMT interference index was calculated with the “TMT b complete time/TMT a complete time” (Golden et al., 1981). The significance threshold was set at 0.05 because we hypothesized that the training would improve the performance of cognitive tests. All analyses were conducted using R Studio.^1^

#### Volumetric Analysis

For the voxel-based morphometry (VBM) analysis, data preprocessing was conducted by using SPM12 (Statistical Parametric Mapping^2^) and CAT12 r1113 (Computational Anatomy Toolbox^3^) implemented in MATLAB R2015a (MathWorks). First, T1-weighted images were segmented by using a fully automated longitudinal segmentation pipeline included in CAT12, which is suitable to detect short-term plasticity effects. The Diffeomorphic Anatomical Registration through an Exponentiated Lie algebra (DARTEL) algorithm was applied to the segmented images to conduct additional optimized spatial normalization for the adolescent brain. Nonlinear deformation parameters were estimated and applied to the pre- and post-training bias-corrected GM tissue images. Finally, the images were smoothed with an 8-mm full-width at half-maximum (FWHM) isotropic Gaussian kernel.

Considering the rapid rate of brain maturation in adolescence (Thompson et al., 2001; Vijayakumar et al., 2014), we conducted group × time repeated-measure ANOVA to examine the presence of training-induced neural changes (Nieuwenhuis et al., 2011). Total intracranial volume was included as a covariate in the model to adjust for global effect. We conducted the analysis based on the liberal threshold (*p* < 0.001 uncorrected) with a cluster of greater than 50 voxels to achieve a reasonable compromise between type 1 and 2 errors. The optimal cluster size threshold was chosen by adjusting the current VBM voxel resolution (2 mm^3^) to a previous study’s suggestion (Lieberman and Cunningham, 2009; Lewis et al., 2011). The regions of interest were brain areas most relevant to the contents of the training program, i.e., the right inferior frontal cortex (IFC), the dorsal anterior cingulate cortex (Aron et al., 2014) and the middle frontal gyrus (Badre and D’Esposito, 2009).

Lastly, the correlation analysis was conducted to identify whether the brain structural changes were associated with the improvement in cognitive performance following training. To conduct the analysis, we extracted the mean value of the smoothed GM density images within the identified cluster where significant time × group effect was observed. We then subtracted the pre-training volume value from the post-training volume value and performed Pearson’s correlation analysis between the changes of both regional GM volume and cognitive test scores.

In addition, correlation analysis was conducted to investigate the relationship between cognitive function changes and GM volume changes at the whole brain level (*p* < 0.001, uncorrected). GM volume change images were calculated by subtracting pre-training images from post-training images of each individual with the ImCalc function in SPM 12. Then, we identified whether the training effect on the regional brain changes overlaps with the brain changes associated with larger cognitive performance gain.

#### Cortical Thickness Analysis

We examined the effect of cognitive training on the cortical thickness by using a fully automated processing surface-based morphometry (SBM) analysis pipeline implemented in CAT12. Since GM volume density highly correlates with other morphological features (i.e., sulcal depth, surface area), cortical thickness analysis was expected to depict more detailed structural change occurring after the training. First, cortical surface reconstruction was conducted based on the projection-based thickness method (Dahnke et al., 2013). And then, topological defects were corrected by using spherical harmonics (Yotter et al., 2011a) which was followed by reparametrizing cortical surface mesh (Yotter et al., 2011b). Surface data was smoothed with 15 mm FWHM isotropic Gaussian kernel. The flexible factorial analysis was conducted to investigate time × group interaction effect for left and right hemisphere respectively. We adopted the same threshold as VBM analysis for exploratory purpose.

## Results

### Behavioral Data

At baseline, scores of the cognitive tests were comparable between the two groups (*p*s > 0.178, Table 2). After 6 weeks of training, the cognitive performance of the training and control groups increased within the group, respectively. The repeated-measure ANOVA to identify the group differences of change in the cognitive scores demonstrated that the Block Design (BD) test performance in the training group was significantly more improved compared to the control group (*p* = 0.007). This interaction effect, however, was not significant after FDR correction (*p* = 0.076).

**Table 2:** Pre- and post-scores and performance changes in the cognitive tests.

	Training Group (*n* = 25)				Control Group (*n* = 28)				Baseline *t*^†^	Group × Time F	η^2^
	Pre	Post	*t*‡	*d*	Pre	Post	*t*‡	*d*			
Cognitive Control											
Stroop	**10.11 (7.22)**	**13.90 (8.46)**	**2**.72*	**0**.**54**	10.37 (6.92)	10.53 (10.66)	0.08	0.02	-0.13	2.15	0.04
TMT	2.35 (0.64)	2.44 (0.83)	0.45	0.09	2.61 (0.70)	2.37 (0.77)	-1.20	-0.23	-1.36	1.34	0.03
Working Memory											
SSP	**27.92 (4.25)**	**31.16 (6.16)**	**2**.43*	**0**.**49**	27.75 (4.33)	28.79 (4.98)	1.65	0.31	0.14	2.39	0.05
DS	32.76 (5.17)	33.60 (5.31)	1.15	0.23	32.32 (4.26)	32.93 (5.13)	0.84	0.16	0.33	0.05	0.00
AR	29.28 (1.75)	29.16 (2.50)	-0.32	-0.06	28.61 (2.33)	28.93 (1.84)	0.83	0.16	1.2	0.67	0.01
Episodic Memory											
VPA1	**46.60 (6.77)**	**52.16 (3.64)**	**5**.98***	**1**.**20**	**48.07 (5.27)**	**53.46 (1.75)**	**5**.63***	**1**.**06**	-0.88	0.02	0.00
VPA2	**13.52 (.87)**	**13.80 (0.50)**	**2**.28*	**0**.**46**	13.71 (0.81)	13.86 (0.59)	0.72	0.14	-0.84	0.33	0.01
DE1	**88.96 (10.27)**	**96.76 (15.76)**	**2**.83**	**0**.**57**	**87.61 (10.61)**	**95.14 (10.35)**	**3**.97***	**0**.**75**	0.47	0.01	0.00
DE2	**80.88 (11.91)**	**87.92 (18.91)**	**2**.87**	**0**.**57**	**79.32 (11.57)**	**87.61 (11.72)**	**4**.93***	**0**.**93**	0.48	0.18	0.00
Intelligence											
BD	**55.56 (7.75)**	**61.48 (4.81)**	**6**.84***	**1**.**37**	55.54 (7.90)	57.68 (6.40)	2.14*	0.40	0.01	7.93**	0.14
MR	28.84 (2.88)	29.44 (2.99)	0.86	0.17	28.54 (2.96)	29.21 (2.43)	1.12	0.21	0.38	0.01	0.00

*Stroop, Stroop interference score; TMT, Trail Making Test interference index; SSP, Symbol Span; DS, Digit Span; AR, Arithmetic; VPA1, Verbal Paired Associate immediate recall; VPA2, Verbal Paired Associated delayed recall; DE1, design immediate recall; DE2, design delayed recall; BD, Block Design; and MR, Matrix Reasoning. ^∗^*p* < 0.05, ^∗∗^*p* < 0.01, ^∗∗∗^*p* < 0.001. Bold represents the significant results after FDR correction at *p* < 0.05. †Independent *t*-test results to assess the group differences in baseline performances. ‡Paired *t*-test results to assess the changes between pre- and post-performance*.

**Figure 1:**
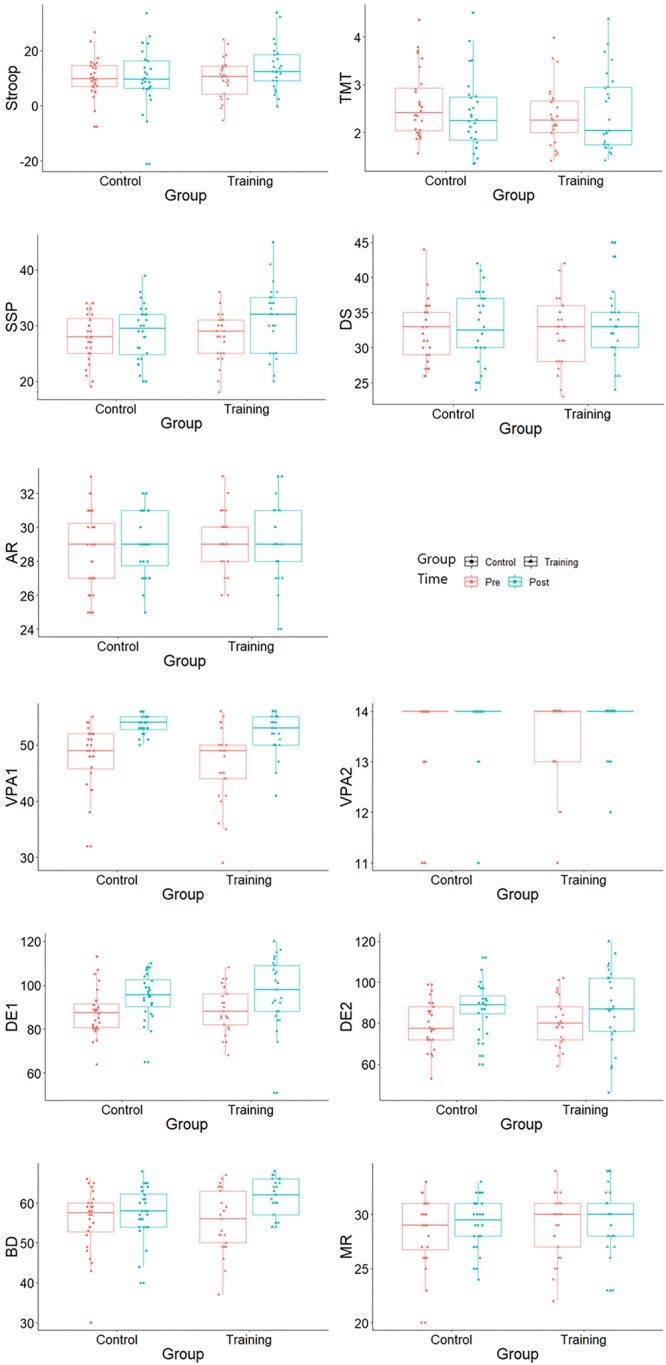
Cognitive test scores at baseline and after training. Stroop, Stroop interference score; TMT, Trail Making Test interference index; SSP, Symbol Span; DS, Digit Span; AR, Arithmetic; VPA1, Verbal Paired Associate immediate recall; VPA2, Verbal Paired Associated delayed recall; DE1, design immediate recall; DE2, design delayed recall; BD, Block Design; and MR, Matrix Reasoning.

Although no time × group interaction effect was observed in the cognitive control, the working memory and the episodic memory performances (*p*s > 0.127), significant improvements within-group in the Stroop (*p* = 0.012), spatial working memory (*p* = 0.023) and verbal memory (*p*s < 0.032) were observed. Cognitive performances at baseline and after-training are both illustrated in Figure 1.

The Stroop and the TMT scores were the interference index and should be interpreted with caution. Since the lower performance in the interference index is affected by the higher performance in the other congruent conditions, a decline of the interference index does not always indicate a general decline in each condition. The Stroop word, the color, the color–word, the TMT A and the TMT B scores all improved after training within group (*p*s < 0.037 for the training group, *p*s < 0.051 for the control group).

### Practice Effects in the Training Tasks

With practice, we expected the difficulty level and accuracy of each task to increase. After the completion of the MTCC protocol, the level of Location–Number Stroop and Counting Clovers upgraded significantly (*t* = 2.72, *p* = 0.013, *t* = 3.89, *p* = 0.004), and the level of Updating (location) showed tendency to increase (*t* = 1.88, *p* = 0.089). In addition, the accuracy of Updating (letter) and Counting Clovers significantly improved (*t* = 4.40, *p* < 0.001, *t* = 3.35, *p* = 0.004), and the accuracy of Location-Number Stroop and Updating (color) demonstrated tendencies of improvement (*t* = 2.05, *p* = 0.057, *t* = 1.82, *p* = 0.095). Training performance of other training tasks did not demonstrate significant increments or improvements (*p*s > 0.104).

### Brain Structural Changes

#### Volumetry

**Figure 2:**
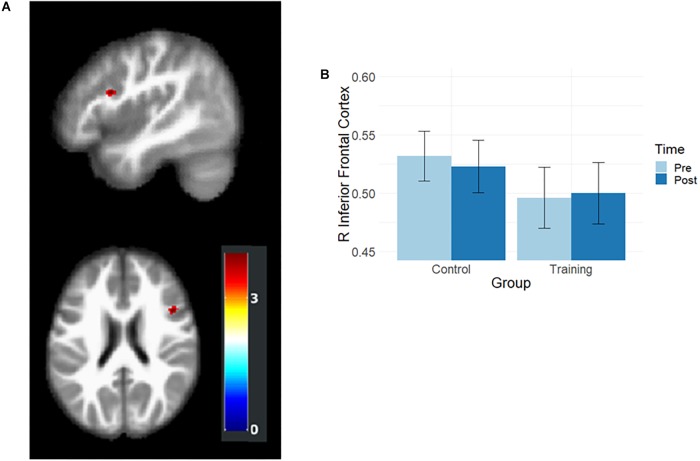
Results of volumetric analysis. **(A)** Interaction effects were observed in rIFC. The map was illustrated at *p* < 0.001 (uncorrected) and *k* > 50. **(B)** The rIFC volume at the pre- and post-training assessments. After training, the rIFC volume increased in the training group and decreased in the control group.

Gray matter volumes after training were examined by using the flexible factorial analysis, and the group and time interaction effect was observed in the right inferior frontal cortex (rIFC; MNI coordination *x* = 45, *y* = 14, *z* = 23, *t* = 3.95, Figure 2A). In the paired *t*-test, the training group showed a slight volume increment (*t* = 2.02, *p* = 0.055), and the control group demonstrated significant volume reduction (*t* = –3.36, *p* = 0.002) in rIFC (Figure 2B).

#### Relationship Between GM Volume and Cognitive Performance Changes

**Figure 3:**
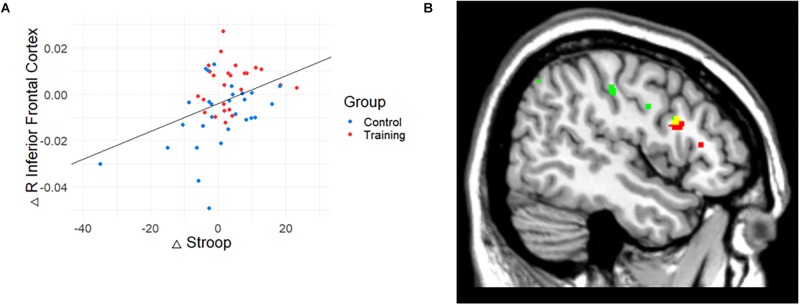
The relationship between gray matter (GM) volume and Stroop performance. **(A)** The relationship between GM volume and cognitive performance. Change in Stroop test performance was positively correlated with the change in the volume of rIFC. **(B)** Conjunction map of training-related regions (red) and Stroop-related regions (green). Yellow represents the overlapping areas. The map was illustrated at *p* < 0.005 (uncorrected) for visualization.

To investigate the meaning of the volumetric change, the association between GM volume and cognitive performance was analyzed (Table 3). The volume change within the rIFC cluster was positively correlated with the change of Stroop (*r* = 0.39, *p* = 0.004, Figure 3A) and VPA2 (*r* = 0.32, *p* = 0.021) performance. After FDR correction, only the correlation with the Stroop test remained significant (*p* = 0.044).

**Table 3:** Pearson’s correlations between the rIFC volume and cognitive performance changes (*N* = 53).

	*r*^a^	*p*^a^	*r*^b^	*p*^b^	*r*^c^	*p*^c^
**Stroop**	**0**.**39**	**0**.**004**	0.15	0.470	0.41	0.034
TMT	-0.03	0.824	-0.13	0.546	-0.14	0.477
SSP	0.20	0.144	0.19	0.366	0.14	0.482
DS	0.14	0.306	0.27	0.195	0.05	0.789
AR	-0.12	0.412	-0.15	0.474	-0.05	0.794
VPA1	0.16	0.251	0.18	0.376	0.14	0.484
VPA2	0.32	0.021	0.42	0.036	0.32	0.101
DE1	0.06	0.663	-0.08	0.708	0.19	0.331
DE2	-0.04	0.771	-0.08	0.711	0.08	0.705
BD	0.15	0.280	-0.19	0.368	0.09	0.656
MR	-0.24	0.082	-0.23	0.279	-0.27	0.173

*Stroop, Stroop interference score; TMT, Trail Making Test interference index; SSP, Symbol Span; DS, Digit Span; AR, Arithmetic; VPA1, Verbal Paired Associate immediate recall; VPA2, Verbal Paired Associated delayed recall; DE1, Design immediate recall; DE2, Design delayed recall; BD, Block Design; and MR, Matrix Reasoning; Bold represents the significant results after FDR correction at *p* < 0.05; a: all participants, b: training group, c: control group*.

Also, correlation analysis between cognitive functions and GM volume changes at the whole brain level showed that Stroop performance was positively associated with a change in GM volume in the right IFC (*p* < 0.001, uncorrected, *k* = 15). While the result did not meet the initial statistical criterion, the homologous region mostly overlapped with the train-related GM volume change (*k* = 13; *x* = 47, *y* = 14, *z* = 24). The conjunction map of training-related regions (red) and Stroop-related regions (green) are shown in Figure 3B (yellow represents the overlapping areas).

#### Cortical Thickness

In addition to volumetry, change in cortical thickness was examined with group × time interaction. The left and right hemispheres were separately analyzed. Cortical thickness of the right hemisphere had an interaction effect in the right inferior frontal cortex (rIFC; MNI coordination *x* = 47, *y* = 12, *z* = 21, *p* < 0.001, uncorrected, *k* = 35), but no interaction was observed in the left cortical thickness. This result was consistent with that of the VBM analysis.

## Discussion

Cognitive control training in normally developing adolescents with multi-component cognitive control tasks resulted in improvement in cognitive function, albeit limited, and volume increase in the right inferior frontal cortex (rIFC). More specifically, significant improvement in a visuospatial fluid intelligence test (Block Design) was observed in the training group compared to the control group, which did not reach significance after the statistical correction was done for multiple comparisons. Compared to the typical developmental reduction of cortical volume in the rIFC observed in the control group, a reversed pattern was evident in the training group. This post-training change in volume of the rIFC was positively correlated with the performance enhancement for inhibitory control. These results suggest that the cognitive training with MTCC has a modest effect on cognitive improvement and yielded morphological change in the brain regions critical to inhibitory control.

It is noteworthy that Block Design (BD) demonstrated the largest gain among the tests used to measure cognitive improvement after the MTCC. The BD test requires visual construction to be conducted parallel to visual reasoning and also involves sustained attention, planning, organization of strategies, error correction, and inhibition of automatic perceptual grouping, most of which overlap with the subcomponents of cognitive control. The enhanced efficiency of the cognitive control processes may have mediated performance improvement in the BD performance of the training group. Consistently, within-group improvement was observed in the Symbol Span (SSP) performance. In fact, most (five out of seven) training tasks required visuospatial working memory, which could have contributed to the improvement in visuospatial test performances. As has been demonstrated elsewhere in the literature, visuospatial working memory is related to spatial ability (Miyake et al., 2001), and working memory training may specifically enhance performance in tasks that tap visuospatial capacity (Colom et al., 2013).

Cognitive training effect tends to have a small or medium effect size, being especially smaller in the untrained domain (Karbach and Kray, 2009; Karbach and Verhaeghen, 2014). While an extensive review of the effect size of the training indicated that the far-transfer effect is generally minimal and smaller than that in the trained domain (i.e., cognitive control) (Sala and Gobet, 2017), our observation showed a relatively larger effect size in the performance of visuospatial intelligence. This may have its origin in the fact that adolescents have highly plastic brains, and their cognitive functions are not fully developed. In addition, cognitive functions are not well segregated at an early age (Wiebe et al., 2008), and the presence of this ambiguous boundary might have contributed to the improvement on the untrained test of general cognition or intelligence. It is possible, however, that this far-transfer effect could stem from the intrinsic nature of the MTCC, such as the multi-component nature of the cognitive control training tasks. This is supported by the previous observation in the elderly population, which found improvement in general cognitive functioning as well as recognition after administration of the MTCC (Kim et al., 2017).

Despite expectations, cognitive control and working memory test performance did not show significantly more improvement in the training group compared to the control group. However, it is notable that the effect size of the Stroop test was comparable to that of near transfer in the previous meta-analysis study (*d* = 0.5) (Karbach and Verhaeghen, 2014). The Stroop test is commonly used to measure the ability to exert cognitive control because it requires the individual to overcome the intrusion of automatic processing in word reading. In the training program, all tasks stimulated inhibitory control (Kim et al., 2017), and repeated practices and activation of the inhibition process may have enhanced the efficiency of the specific cognitive control process, possibly resulting in some improvement on the Stroop test.

Morphological responsiveness of the gray matter (GM) to the cognitive training showed that the training induced change was significant for one of the key brain regions involved in response inhibition, namely, the right inferior frontal cortex (rIFC). Interestingly, adolescents who did not receive the full version of the MTCC showed a steep decrease in GM volume in this region, while this trajectory was reversed in the training group with relative increase in its volume. According to the literature, rIFC is an important region for the execution of inhibition (Aron et al., 2004, 2014). Brain lesions and neuroimaging studies have highlighted the critical role of the rIFC in performing inhibition tasks (Aron et al., 2003; Chikazoe et al., 2007; Madsen et al., 2010). The MTCC was designed to enhance the performance of cognitive control, and it contained all major components of cognitive control (i.e., inhibition, updating, and shifting), while all these subcomponents correlated highly with one another, due to a common factor involving inhibition (Friedman and Miyake, 2017). Therefore, it is possible that the MTCC effectively changed the GM microstructure, especially the part that is relevant to the common inhibitory process. Further, our results are consistent with previous studies that found similar structural and functional changes following inhibitory control training (Berkman et al., 2014; Chavan et al., 2015).

The relative increase in gray matter volume following training, however, appears somewhat counterintuitive because it acted against the typical trajectory of morphological development (Klein et al., 2014; Giedd et al., 2015). Two explanations are possible. First, a developmental decrease in the volume of GM generally reflects the neurobiology of pruning, but several studies have indicated that accelerated pruning occurring in cortical synaptic density during adolescence may not always reflect optimal development and may furthermore be related to abnormal neurodevelopment and possibly indicate loss of synapses necessary for neurocognitive function (Chung et al., 2018). Further, it has also been reported that adolescents with a more enriched environment (e.g., higher family income and parental education) showed delayed age-related decreases in GM volume of the inferior frontal cortex (Noble et al., 2012). Another possibility is that experience-dependent morphological change may indicate heterogeneous cellular mechanisms that are not directly associated with the pruning process (e.g., dendritic branching or angiogenesis) (Zatorre et al., 2012). Although previous studies have reviewed the possibility that cognitive training accelerates the ongoing trajectory of brain development (Jolles et al., 2012), our results show that repetitive cognitive training affects brain morphology through mechanisms that may be independent of the pruning process or moderating the pruning process. This interpretation is consistent with the results of a previous study showing that participants who underwent working memory training showed relative conservation in rIFC thickness, whereas the control group showed large cortical thinning in young women (Román et al., 2015).

While rapid developmental change in the brain regions after cognitive training is a well-known phenomenon, there has been little discussion of how experience-dependent structural change can be detected alongside typical brain development. Surprisingly, many previous studies examining the neural effects of cognitive training in the maturing adolescents have omitted the control group (Jolles et al., 2010, 2012, 2013, 2016), whose results might have been heavily confounded by the ongoing brain development. Even when no explicit neural changes were observed within the training group, more detailed scrutiny comparing it with a control group is necessary to distinguish training-specific effects versus the typical neurodevelopmental change. Consistently, the training effect on cortical thickness were similar to that on the GM volume. Since this measure is more sensitive to volume change *per se* than the volumetry, it further supports the thickening of the gray matter.

Parallel changes in cognitive control and gray matter volume following the MTCC were observed between Stroop performance and the rIFC. This is consistent with what was found in previous studies that found close link between cortical thickness in the rIFC or its adjacent regions and the performance of inhibition tasks, and impulsivity-related behavior disorders, such as ADHD (Odlaug et al., 2014; Newman et al., 2016; Berner et al., 2018). Therefore, the gray matter volume change may be positively associated with the change in Stroop performance. Cautious interpretation, however, is needed since the major source of association between brain and cognitive change was due to the volume reduction in the control group in our study. It is possible that the training effect may have counteracted the developmental GM volume decrease, and the narrow change range of rIFC volume in the training group might have limited its correlation with Stroop performance change.

Limitations of the study should be mentioned. First, we were not able to observe significantly improved cognitive outcomes in the training condition relative to the control group in the near-transfer domains: inhibitory control and working memory. Although our additional analysis supplemented our indirect interpretation, further research is required to determine the results in the near-transfer effect. For their part, individuals with high performance were already using an efficient strategy and have less room for improvement, according to the compensation view (Lövdén et al., 2012). Most participants in this study had above-average intelligence (84th–99th percentiles, using the scaled score for the Vocabulary intelligence subtest), which supports a previous study that found that the cognitive training effect on brain morphology was only present in individuals with relatively low intelligence (Román et al., 2015).

While some effect sizes similar to those found in previous studies (*d* = 0.5) were observed here, the use of scores with contrasting methods (subtracting the additional interference effect) is highly prone to low reliability, and this may have degraded the sensitivity of detection of training effects (Hedge et al., 2018; Enkavi et al., 2019). In addition, the sample size was too small to convincingly demonstrate training effects. Future research with larger sample size is necessary to confirm our findings.

Another caveat was that although we identified changes in GM, the exact underlying cellular and molecular level changes remain unknown, and our interpretation is limited. Volume or thickness changes in the GM reflect various candidate mechanisms that contribute to complex behavioral changes (Zatorre et al., 2012). The structural imaging method we employed was limited in disambiguating the specific meaning of the GM changes. For instance, it is possible that the relative increase in GM volume can be explained with the expansion–renormalization model (Wenger et al., 2017). The model suggests that the GM volume of the task-relevant region increases at the beginning of learning, stabilizing in the later phases through cellular processes that include neurogenesis, synaptic changes, dendritic branching, axon sprouting, and changes in glial number and morphology. It also suggests that the observed effects of training on the GM volume can differ depending on the phase of training. In our study, the training might have induced changes in the cellular processes in the initial stage and was observed as relative conservation in GM. Since investigation with structural MRI alone cannot identify neural changes exactly, further research is necessary to elucidate the exact neural mechanisms of change in the cognitive control network after training.

## Conclusion

In conclusion, cognitive control training that utilized multiple subcomponents improved visuospatial intelligence with limitation in normally developing adolescents, while it relative increased their right inferior frontal cortex volume, a key brain region for inhibitory control. The multi-component training involved all the subcomponents of cognitive control, such as inhibition, working memory, shifting, and dual tasking, which may have resulted in some improvement in adolescents’ fluid intelligence. By including an active control group, we were able to differentiate developmental change and the Hawthorne effect from the true training effects, and found the reversed developmental trajectory of volume increase in the rIFC in the training group. These findings may have implications and applications in typically developing adolescents as well as psychiatric patients who have been found to have excessive pruning during adolescence, such as those with schizophrenia.

## Ethics Statement

This study was carried out in accordance with the recommendations of the Institutional Review Board of Seoul National University with written informed consent from all subjects. All subjects gave written informed consent in the presence of legal guardians and in accordance with the Declaration of Helsinki. The protocol was approved by the Institutional Review Board of Seoul National University.

## Author Contributions

DL and JC contributed to conception and the design of the study. DL organized the database and wrote the first draft of the manuscript. DL and SK performed the statistical analysis and wrote sections of the manuscript. JC wrote the final revision. All authors contributed to the manuscript revision, and read and approved the submitted version.

## Conflict of Interest Statement

JC initiated a collaborative project funded by the 433 Inc., the mobile game company which was interested in mobile cognitive training programs. Development of the cognitive training program, recruitment of research participants, and administration and analysis of experiments were conducted independently from the 433 Inc. The remaining authors declare that the research was conducted in the absence of any commercial or financial relationships that could be construed as a potential conflict of interest.

## Footnotes

1 www.rstudio.com

2 www.fil.ion.ucl.ac.uk/spm/software/spm12

3 http://dbm.neuro.uni-jena.de/cat/
